# The Effect of 2-Ketobutyrate on Mitochondrial Substrate-Level Phosphorylation

**DOI:** 10.1007/s11064-019-02759-8

**Published:** 2019-02-27

**Authors:** David Bui, Dora Ravasz, Christos Chinopoulos

**Affiliations:** Department of Medical Biochemistry, Semmelweis University, Tuzolto st. 37-47, Budapest, 1094 Hungary

**Keywords:** Alpha-ketobutyrate, 2-Oxobutyrate, 2-Oxobutanoate, Succinyl-CoA

## Abstract

The reaction catalyzed by succinate-CoA ligase in the mitochondrial matrix yields a high-energy phosphate when operating towards hydrolysis of the thioester bond of succinyl-CoA, known as mitochondrial substrate-level phosphorylation (mSLP). The catabolism of several metabolites converge to succinyl-CoA but through different biochemical pathways. Among them, threonine, serine and methionine catabolize to succinyl-CoA through the common intermediate, 2-ketobutyrate. During the course of this pathway 2-ketobutyrate will become succinyl-CoA through propionyl-CoA catabolism, obligatorily passing through an ATP-consuming step substantiated by propionyl-CoA carboxylase. Here, by recording the directionality of the adenine nucleotide translocase while measuring membrane potential we tested the hypothesis that catabolism of 2-ketobutyrate negates mSLP due to the ATP-consuming propionyl-CoA carboxylase step in rotenone-treated, isolated mouse liver and brain mitochondria. 2-Ketobutyrate produced a less negative membrane potential compared to NADH or FADH_2_-linked substrates, which was sensitive to inhibition by rotenone, atpenin and arsenate, implying the involvement of complex I, complex II and a dehydrogenase—most likely branched chain keto-acid dehydrogenase, respectively. Co-addition of 2-ketobutyrate with NADH- or FADH_2_-linked substrates yielded no greater membrane potential than in the presence of substrates alone. However, in the presence of NADH-linked substrates, 2-ketobutyrate prevented mSLP in a dose-dependent manner. Our results imply that despite that 2-ketobutyrate leads to succinyl-CoA formation, obligatory metabolism through propionyl-CoA carboxylase associated with ATP expenditure abolishes mSLP. The provision of metabolites converging to 2-ketobutyrate may be a useful way for manipulating mSLP without using pharmacological or genetic tools.

## Introduction

**Fig. 1:**
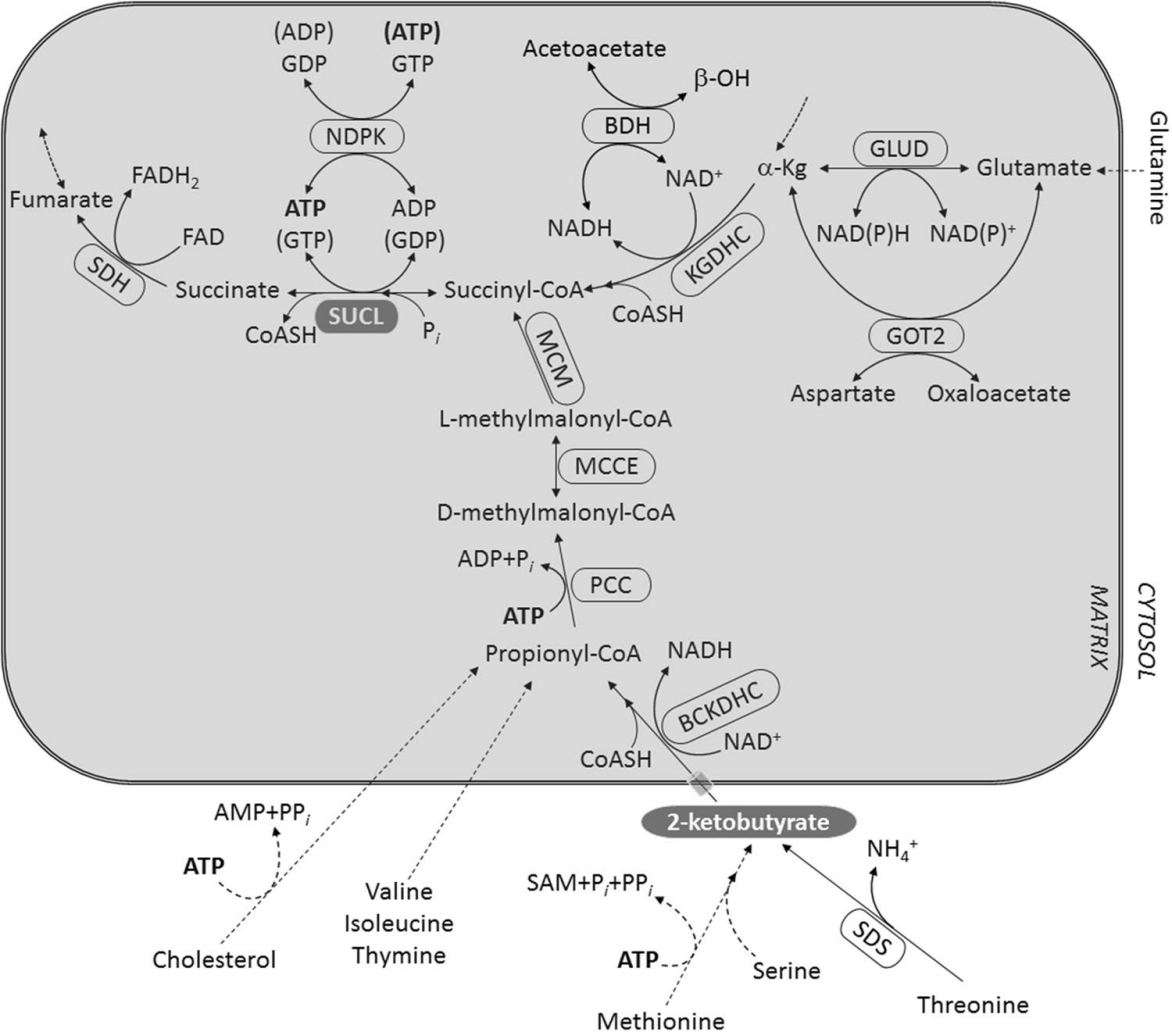
Catabolism of metabolites towards succinyl-CoA. BCKDHC: branched-chain keto-acid dehydrogenase; BDH: β-hydroxybutyrate dehydrogenase; GLUD: glutamate dehydrogenase; GOT2: aspartate aminotransferase; KGDHC: ketoglutarate dehydrogenase complex; MCM: methylmalonyl mutase; MCEE: methylmalonyl racemase; NDPK: nucleoside diphosphokinase; PCC: propionyl-CoA carboxylase; SAM: S-adenosylmethionine; SDH: succinate dehydrogenase; SDS: l-serine dehydratase/l-threonine deaminase; SUCL: succinate-coA ligase. Dashed arrows imply multiple steps which may occur inside or outside the mitochondrial matrix. Entrance of 2-KB into the matrix likely occurs through the mitochondrial pyruvate carrier (depicted by a grey semi-transparent cylinder)

Mitochondrial substrate-level phosphorylation (mSLP) mediated by succinate-CoA ligase is a reversible process by which ATP (or GTP, depending on subunit composition of the enzyme [1, 2]) can be generated in the absence of oxidative phosphorylation. This is feasible due to the high energy stored in the thioester bond of succinyl-CoA. A number of metabolites converge towards succinyl-CoA; however, with the exception of those catabolizing first through α-ketoglutarate, all others will obligatorily pass through biochemical pathways encompassing at least one ATP-expenditure step, see Fig. 1. Catabolism of threonine and methionine converge to 2-ketobutyrate (2-KB, also known as α-ketobutyrate, 2-oxobutyrate, 2-oxobutanoate, CAS Registry Number: 600-18-0), prior to entering into the propionate catabolic pathway towards succinyl-CoA. Serine joins the methionine catabolic pathway by combining with homocysteine forming cystathionine which forms 2-KB, cysteine and ammonia by cystathionine gamma-lyase. All of the reactions leading to 2-KB formation occur outside mitochondria, thus 2-KB entry into the matrix for subsequent catabolism is warranted. Mindful that in the absence of oxidative phosphorylation the directionality of the adenine nucleotide translocase (ANT) and the reaction catalyzed by succinate-CoA ligase are in directional synchrony [3] linked by the matrix [ATP]/[ADP] [4, 5], we hypothesized that metabolites converging to succinyl-CoA through ATP-consuming pathways would negate mSLP, and this would be reflected in the reversal of ANT when the electron transport chain is inhibited. Part of this work has been published before in abstract form [6].

## Materials and Methods

### Animals

Mice were of mixed 129 Sv and C57Bl/6 background. The animals used in our study were of either sex and between 2 and 6 months of age. Data obtained from liver mitochondria of mice of a particular gender or age (2, 4 or 6 months) did not yield any qualitative differences, thus all data were pooled. Mice were housed in a room maintained at 20–22 °C on a 12-h light–dark cycle with food and water available ad libitum. All experiments were approved by the Animal Care and Use Committee of the Semmelweis University (Egyetemi Állatkísérleti Bizottság).

### Isolation of Mitochondria

Liver and brain mitochondria were isolated as described in Ref. [7]. Protein concentration was determined using the bicinchoninic acid assay, and calibrated using bovine serum standards [8] using a Tecan Infinite® 200 PRO series plate reader (Tecan Deutschland GmbH, Crailsheim, Germany).

### Determination of Membrane Potential in Isolated Mitochondria

ΔΨm of isolated mitochondria (0.5 mg for mouse liver and 0.25 mg for brain per 2 ml of medium) was estimated fluorimetrically with safranine O [9], acknowledging the considerations elaborated in [10, 11] regarding inhibition of respiration as well as unspecific binding of safranine. Fluorescence was recorded in a Hitachi F-7000 spectrofluorimeter (Hitachi High Technologies, Maidenhead, UK) at a 5-Hz acquisition rate, using 495- and 585-nm excitation and emission wavelengths, respectively. Experiments were performed at 37 °C.

Reagents: Standard laboratory chemicals and 2-ketobutyrate (Cat. No.: K401, purity 97%) were from Sigma. SF 6847 and atpenin A5 were from Enzo Life Sciences (ELS AG, Lausen, Switzerland). Carboxyatractyloside (cATR) was from Merck (Merck KGaA, Darmstadt, Germany). Mitochondrial substrate stock solutions were dissolved in bi-distilled water and titrated to pH 7.0 with KOH. ADP was purchased as a K^+^ salt of the highest purity available (Merck) and titrated to pH 6.9.

## Results

### Catabolism of Metabolites Towards Succinyl-CoA

As shown in Fig. 1, a number of metabolites converge to succinyl-CoA such as glutamine, threonine, serine, methionine, valine, isoleucine, thymine, cholesterol and of course others originating upstream from α-ketoglutarate. Dashed arrows imply multiple steps occurring in either inside or outside the mitochondrial matrix. Catabolism of threonine, serine and methionine lead to 2-KB generation which would enter the mitochondrial matrix and get converted to propionyl-CoA by the branched-chain keto-acid dehydrogenase complex (BCKDHC), and then subsequently to d-methylmalonyl-CoA by propionyl-CoA carboxylase (PCC), consuming ATP. In turn, d-methylmalonyl-CoA racemizes to l-methylmalonyl-CoA by methylmalonyl-CoA epimerase (MCEE) and then isomerizes to succinyl-CoA by methylmalonyl-CoA mutase (MCM), a B_12_-dependent enzyme. The aforementioned enzymes reside inside the mitochondrial matrix, and since they process 2-KB it means that this metabolite traverses the inner mitochondrial membrane. To date, a 2-KB-specific carrier has not been identified, though it is known to compete for pyruvate transport through the mitochondrial pyruvate carrier [12–14] and probably the newly identified choline carrier [15, 16]. The reaction catalyzed by β-hydroxybutyrate dehydrogenase (BDH) is also shown, demonstrating the competition between this enzyme complex and KGDHC for NAD^+^, in the presence of excess β-hydroxybutyrate. This phenomenon is exploited in our experimental settings in order to titrate the contribution of KGDHC yielding a-ketoglutarate in mitochondria with an inhibited respiratory chain for the purpose of mSLP. From the above metabolic considerations, we set to investigate if—and to what extent—2-KB serves as a fuel for mitochondria, and if so, does it impact on mSLP.

### 2-KB Supports Generation of ΔΨm in Isolated Liver and Brain Mitochondria by Generating Both NADH and FADH_2_

**Fig. 2:**
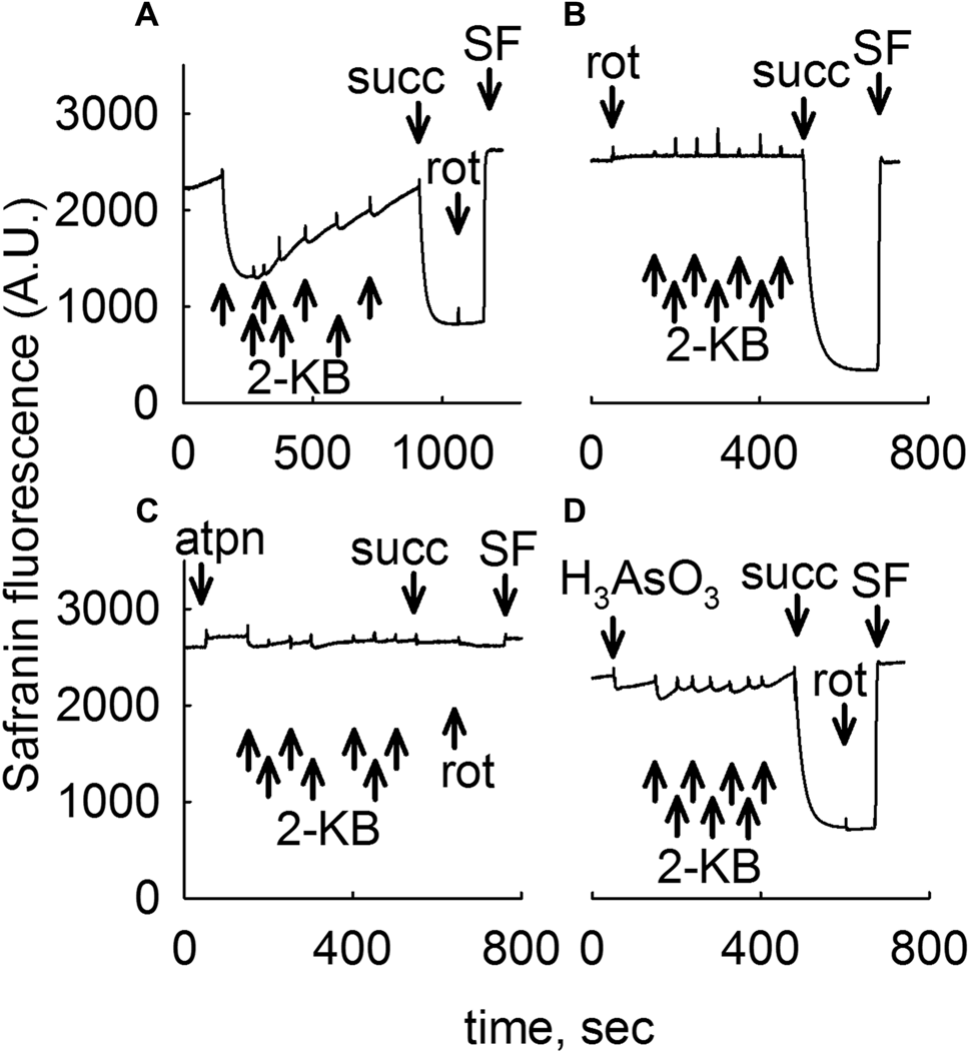
2-KB as a metabolic fuel in mouse liver mitochondria. Reconstructed time courses of safranine O signal in isolated mouse liver mitochondria. The effect of 2-KB pulses (indicated by arrows signifying 0.5 mM each, thus a total of 3.5 mM 2-KB added) is shown. Whenever indicated, succinate (5 mM) or rotenone (1 µM) or atpenin (atpn 1 µM), or arsenite (H_3_AsO_3_, 1 mM) was added. At the end of each experiment 250 nM SF 6847 was added to achieve complete depolarization (an increase in safranine O fluorescence signal implies depolarization). Wherever single graphs are presented, they are representative of at least 4 independent experiments

**Fig. 3:**
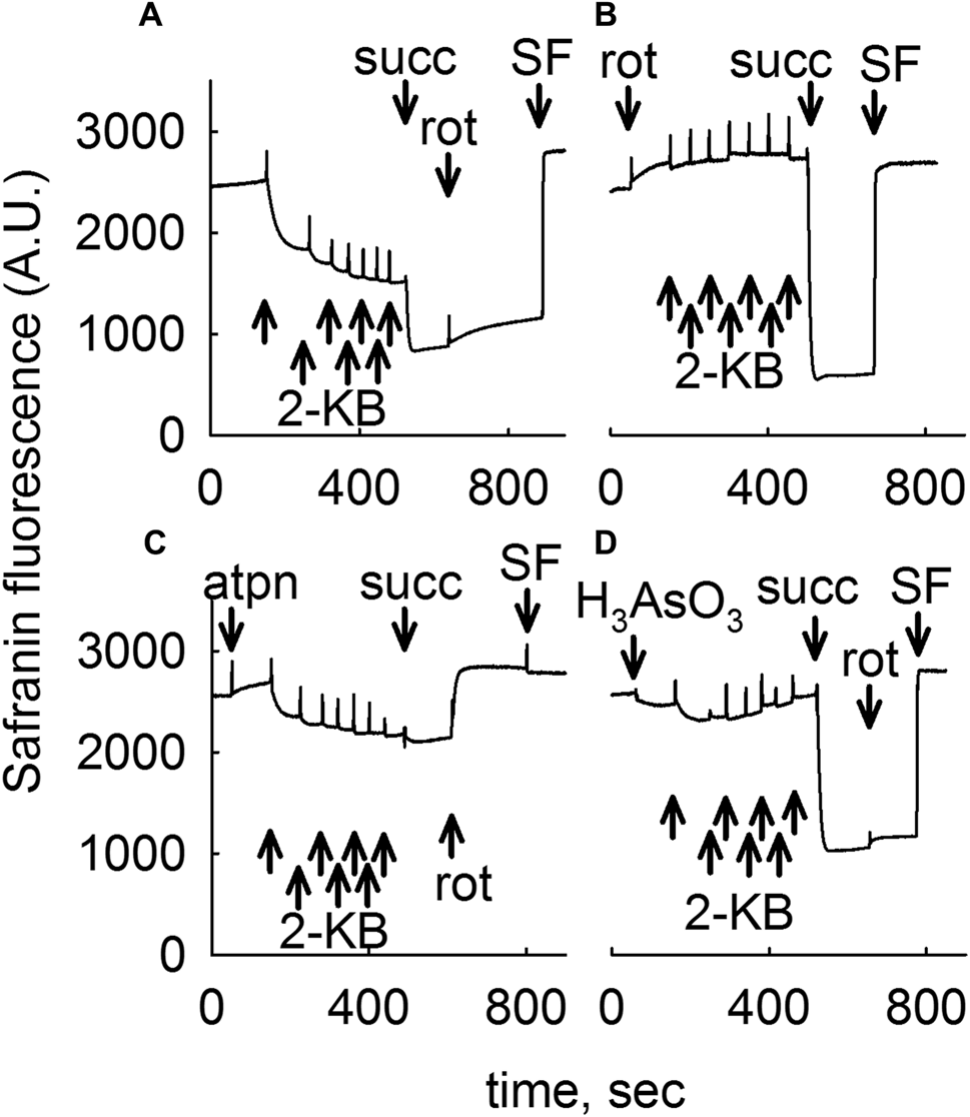
2-KB as a metabolic fuel in mouse brain mitochondria. Reconstructed time courses of safranine O signal in isolated mouse brain mitochondria. The effect of 2-KB pulses (indicated by arrows signifying 0.5 mM each, thus a total of 3.5 mM 2-KB added) is shown. Whenever indicated, succinate (5 mM) or rotenone (1 µM) or atpenin (atpn 1 µM), or arsenite (H_3_AsO_3_, 1 mM) was added. At the end of each experiment 250 nM SF 6847 was added to achieve complete depolarization (an increase in safranine O fluorescence signal implies depolarization). Wherever single graphs are presented, they are representative of at least 4 independent experiments

As shown in Figs. 2 and 3 for liver and brain mitochondria, respectively, addition of seven 0.5 mM 2-KB pulses to mitochondria totaling 3.5 mM (panel Figs. 2a, 3a) conferred a moderate decrease in safranine O fluorescence implying development of ΔΨm. In liver mitochondria, ΔΨm was afterwards becoming gradually lost, as opposed to ΔΨm in brain mitochondria that remained stable. Subsequent addition of succinate (succ, 5 mM) led to maximum polarization. Further addition of the complex I inhibitor rotenone (rot, 1 µM) yielded no (liver mitochondria) or a very small depolarization implying intact operation of complexes III and IV. At the end of the experiment the uncoupler SF 6847 (SF, 250 nM) was added in order to achieve a complete loss of ΔΨm indicating maximum safranine O fluorescence. However, addition of rotenone (Figs. 2b, 3b for liver and brain, respectively), or the complex II inhibitor atpenin (atpn, 1 µM, Figs. 2c, 3c for liver and brain, respectively) or the dehydrogenases inhibitor arsenite (H_3_AsO_3_ 1 mM, Figs. 2d, 3d for liver and brain, respectively) abolished the effect of 2-KB conferring ΔΨm to mitochondria (with the exception of atpenin in brain mitochondria, where a mild decrease in safranine O fluorescence was observed upon addition of 2-KB, Fig. 3c). As expected, subsequent addition of succinate (succ) led to development of ΔΨm if mitochondria were challenged by rotenone or arsenite, but not atpenin. Note that prior to the addition of any substrates liver mitochondria exhibit an initial, transient polarization which is attributed to consumption of endogenous substrates, most likely acyl carnitines. This transient depolarization proceeds to a complete loss of ΔΨm within ~ 1 h. From the above results we deduced that 2-KB affords ΔΨm to mitochondria by mechanisms involving both complex I (fueled by NADH, some originating from BCKDHC, an arsenite-sensitive enzyme complex) and complex II (fueled by FADH_2_).

### 2-KB Abolishes mSLP Conferred by Glutamate or Pyruvate

**Fig. 4:**
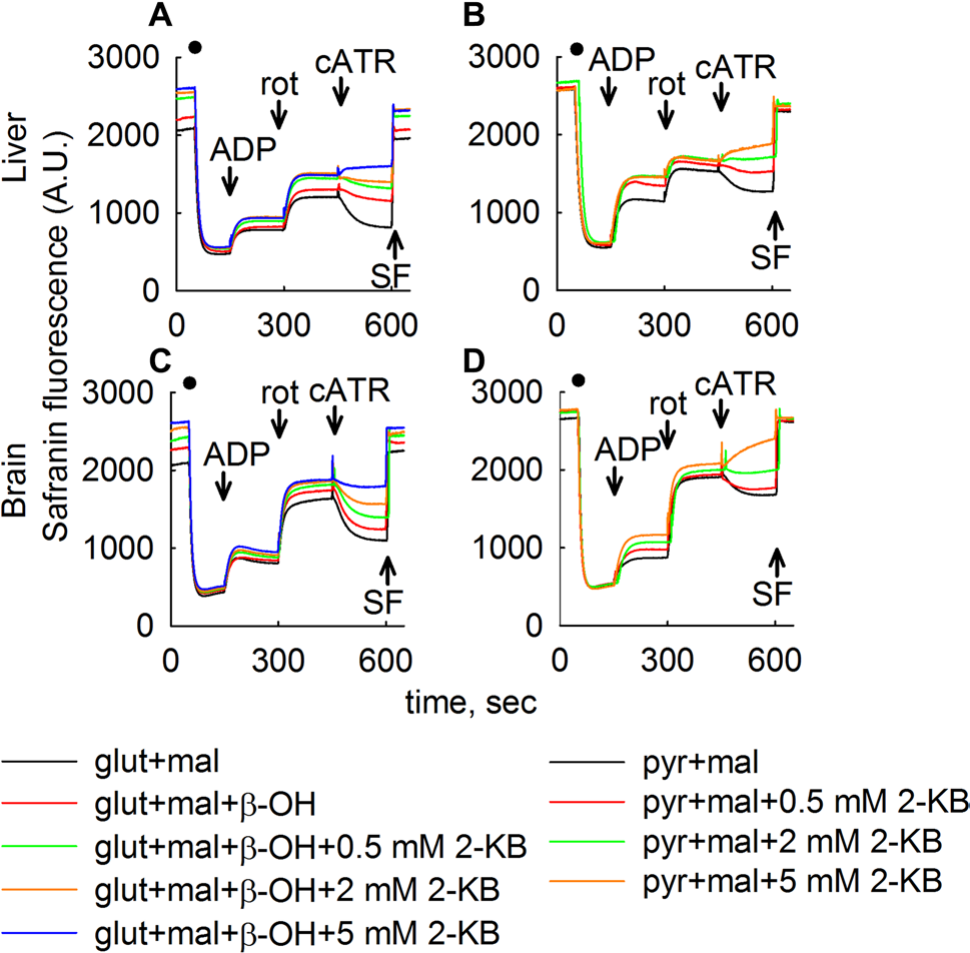
2-KB abolishes mSLP. Reconstructed time courses of safranine O signal in isolated mouse liver (**a**, **b**), or brain (**c**, **d**) mitochondria. Mitochondria were added where indicated by the closed circles. ADP (2 mM) was added where indicated. The effect of cATR (2 µM) on ΔΨm treated with rotenone (rot, 1 µM) in the absence or dose-dependent presence of 2-KB as indicated in the legends is shown. Control traces are shown in black. At the end of each experiment 250 nM SF 6847 was added to achieve complete depolarization (an increase in safranine O fluorescence signal implies depolarization). Wherever single graphs are presented, they are representative of at least four independent experiments

mSLP was addressed by interrogating the directionality of the ANT in mitochondria with an inhibited respiratory chain [3], isolated from mouse liver (Fig. 4a, b) or brain (Fig. 4c, d). The method is based on the electrogenic character of the ANT, as described in [3]; briefly, abolition of a “forward-operating” ANT by carboxyatractyloside leads to a depolarization, while abolition of a reverse-operating ANT leads to repolarization. As shown in Fig. 4, mitochondria were added where indicated by the closed circles and safranine O fluorescence was recorded reflecting ΔΨm. ADP (2 mM) was added where indicated, initiating respiration and causing a mild depolarization due to forward ANT and F_o_–F_1_ ATP synthase operation. Subsequently, oxidative phosphorylation was halted by inhibiting complex I with rotenone (rot). This led to a further loss of ΔΨm; ΔΨm was now maintained by a reverse-function of F_o_–F_1_ ATP synthase. Mindful of the directional synchrony of ANT with SUCL reaction [4], Inhibition of the ANT by carboxyatractyloside hints on the presence of mSLP: repolarization implies that SUCL operates towards ATP (or GTP) formation thus supporting mSLP, while cATR-induced depolarization means that SUCL was hydrolyzing ATP (or GTP). Substrates were either glutamate and malate (5 mM each) or glutamate and malate and 10 mM β-hydroxybutyrate (β-OH) (Fig. 4a, c) or pyruvate and malate (5 mM each) as indicated in the legends of Fig. 4. Dose-dependent addition of 2-KB (0.5, 2 or 5 mM) converted the cATR-induced changes in safranine O fluorescence from a repolarization to a depolarization, implying dose-dependent inhibition of mSLP by 2-KB (which was complete at 5 mM, or 2 mM if β-hydroxybutyrate was also present). This concentration range of 2-KB was chosen for interrogating mSLP mindful of that used in Figs. 2, 3 verifying catabolism of this metabolite. At the end of each experiment, the uncoupler SF 6847 SF, 250 nM was added to achieve a completely depolarized state.

## Discussion

The most important observation of the present work is the abolition of mSLP by 2-KB in isolated mitochondria with an inhibited respiratory chain. This is likely attributed to ATP expenditure by PCC, even though 2-KB supports succinate-CoA ligase by providing succinyl-CoA. As a word of caution though, the effect of BCKDHC stealing NAD^+^ from KGDHC (when glutamate and malate were the main fuels) could also contribute to abrogation of mSLP. The decrease in NAD^+^ provision to KGDHC with the aim of diminishing mSLP is the strategy followed by including β-hydroxybutyrate in the media (supporting NADH generation by β-hydroxybutyrate dehydrogenase) relying on the fact that KGDHC activity is important for mSLP [7]. Provision of NAD^+^ to KGDHC in the absence of oxidative phosphorylation occurs through mitochondrially-localized diaphorases, [17] such as mitochondrially-localized NQO1 [18]. The effect of 2-KB inhibiting pyruvate oxidation has been published before [19], albeit this was only observed at very high 2-KB concentrations (20 mM). This was attributed to a weak inhibitory action of 2-KB on the pyruvate dehydrogenase complex [20, 21].

Our work identifies 2-KB (or metabolites converging towards this molecule) as abrogating mSLP in a physiological manner. In human neutrophils, value ranges of 0.01–0.07 fmol/cell have been reported [22]; mindful that the volume of a human neutrophil is ~ 299 µm^3^ [23], 2-KB cytosolic concentration must be 0.033–0.232 mM. In blood plasma and urine, 2-KB concentration is < 0.01 mM but can be elevated ten- or hundred-fold in certain disease states [24–26], thus, the flux of 2-KB production by several metabolites may indeed reach a threshold upon which mSLP is affected. This can be exploited in a number of metabolic settings in order to interrogate mSLP as part of the citric acid cycle [27], further benefitted by it being membrane-permeable. However, it must be emphasized that 2-KB is also a substrate for lactate dehydrogenase being converted to α-hydroxybutyrate [28], thus, the effect of 2-KB in whole cells or tissues may not only be attributed to the mechanisms outlined in the present study. By the same token, the effects of 2-KB described in [28] could be partially ascribed to its ability of negating mSLP.

Finally, since the catabolism of other metabolites converging to succinyl-CoA (except those originating from α-Kg) besides threonine, serine and methionine also require expenditure of one (such as in case of valine, isoleucine, thymine) or two (such as with cholesterol) molecules of ATP, it is expected that they will also lead to abolition of mSLP. The effect of catabolism of these latter metabolites on mitochondrial ATP output with a dysfunctional respiratory chain is currently under investigation. Relevant to this, it is important to emphasize that although ATP production mediated by succinyl-CoA ligase is small compared to that produced by oxidative phosphorylation, mSLP assumes a critical role in preventing anoxic mitochondria from becoming cytosolic ATP consumers, avoiding straining of glycolytic ATP reserves [3].
